# Coral Energy Reserves and Calcification in a High-CO_2_ World at Two Temperatures

**DOI:** 10.1371/journal.pone.0075049

**Published:** 2013-10-11

**Authors:** Verena Schoepf, Andréa G. Grottoli, Mark E. Warner, Wei-Jun Cai, Todd F. Melman, Kenneth D. Hoadley, D. Tye Pettay, Xinping Hu, Qian Li, Hui Xu, Yongchen Wang, Yohei Matsui, Justin H. Baumann

**Affiliations:** 1 School of Earth Sciences, The Ohio State University, Columbus, Ohio, United States of America; 2 School of Marine Science and Policy, University of Delaware, Lewes, Delaware, United States of America; 3 Department of Marine Sciences, University of Georgia, Athens, Georgia, United States of America; 4 Reef Systems Coral Farm, New Albany, Ohio, United States of America; Leibniz Center for Tropical Marine Ecology, Germany

## Abstract

Rising atmospheric CO_2_ concentrations threaten coral reefs globally by causing ocean acidification (OA) and warming. Yet, the combined effects of elevated *p*CO_2_ and temperature on coral physiology and resilience remain poorly understood. While coral calcification and energy reserves are important health indicators, no studies to date have measured energy reserve pools (i.e., lipid, protein, and carbohydrate) together with calcification under OA conditions under different temperature scenarios. Four coral species, *Acropora millepora, Montipora monasteriata, Pocillopora damicornis, Turbinaria reniformis*, were reared under a total of six conditions for 3.5 weeks, representing three *p*CO_2_ levels (382, 607, 741 µatm), and two temperature regimes (26.5, 29.0°C) within each *p*CO_2_ level. After one month under experimental conditions, only *A. millepora* decreased calcification (−53%) in response to seawater *p*CO_2_ expected by the end of this century, whereas the other three species maintained calcification rates even when both *p*CO_2_ and temperature were elevated. Coral energy reserves showed mixed responses to elevated *p*CO_2_ and temperature, and were either unaffected or displayed nonlinear responses with both the lowest and highest concentrations often observed at the mid-*p*CO_2_ level of 607 µatm. Biweekly feeding may have helped corals maintain calcification rates and energy reserves under these conditions. Temperature often modulated the response of many aspects of coral physiology to OA, and both mitigated and worsened *p*CO_2_ effects. This demonstrates for the first time that coral energy reserves are generally not metabolized to sustain calcification under OA, which has important implications for coral health and bleaching resilience in a high-CO_2_ world. Overall, these findings suggest that some corals could be more resistant to simultaneously warming and acidifying oceans than previously expected.

## Introduction

Anthropogenic climate change threatens many marine ecosystems today, and coral reefs are among the most sensitive to current changes in ocean biogeochemistry [1], [2]. Rising atmospheric carbon dioxide (CO_2_) concentrations have already caused an increase of 0.6°C in the average temperature of the upper layers of the ocean over the past 100 years [3], and about one third of all anthropogenic CO_2_ has been absorbed by the ocean, causing ocean acidification (OA) [4], [5]. Since scleractinian corals are calcifying organisms that already live close to their upper thermal tolerance limits [6], both ocean warming and acidification severely threaten their survival and role as reef ecosystem engineers [1], [7].

The uptake of anthropogenic CO_2_ by the ocean changes the carbonate chemistry of seawater by increasing proton (H^+^) and bicarbonate (HCO_3_^−^) concentrations, while at the same time decreasing the concentration of carbonate (CO_3_^2−^). Consequently, seawater pH (i.e., −log[H^+^]) and the saturation state with respect to aragonite (Ω_arag_ = [Ca^2+^][CO_3_^2−^]/K_sp_ with K_sp_ being the ionic product of [Ca^2+^] and [CO_3_^2−^] under solution-mineral equilibrium) decreases. As aragonite is the form of calcium carbonate (CaCO_3_) precipitated by modern corals, this process compromises marine calcification [8]–[10]. Over the past century, Ω_arag_ in the tropics has decreased from 4.6 to 4.0 [8] and is expected to decrease to 2.5–3.0 by the year 2100 [1], [8], [11]. Further, it has been estimated that scleractinian calcification rates may drop by up to 35%–40% by the end of this century [8], [12].

Coral calcification typically decreases in response to experimentally reduced seawater pH [13]–[24] but not always [14], [15], [25]–[31]. Seawater temperature also influences calcification [32]–[36], resulting in potentially interactive effects of temperature and OA on coral calcification. For example, negative effects of elevated seawater *p*CO_2_ on calcification are often exacerbated when temperature is simultaneously increased [20], [27], [30], suggesting a synergistic interactive effect. However, this is not always observed [19], [28], [31] and in one study even the opposite was shown [37]. Clearly, further studies are required to gain a better understanding of the interactive effects of elevated temperature and *p*CO_2_ on coral calcification and its resistance to OA.

Much less is known about how combined OA and warming will influence other aspects of coral physiology such as energy reserves and tissue biomass. If calcification becomes energetically more costly under elevated *p*CO_2_ due to a decreased aragonite saturation state [38]–[40], then the extra energy needed to maintain calcification might be drawn from one or more of the following sources: 1) Coral energy reserves (i.e., lipids, protein, carbohydrates), 2) Enhanced endosymbiotic algal production due to CO_2_ fertilization [41], and 3) Increased heterotrophy (i.e., zooplankton, particulate and/or dissolved organic carbon) [28], [42]. These responses may be even more extreme with the simultaneous increases in seawater temperature because tissue biomass, energy reserves, and endosymbiotic algal density are typically lowest when temperature (and irradiance) is highest on seasonal timescales [43], [44], [45] and under bleaching scenarios [46], [47], [48].

Although tissue biomass and energy reserves are important indicators of coral health [46], [47] and play a significant role in promoting resilience to bleaching [49], no studies to date have measured all three energy reserve pools (i.e., lipid, protein, and carbohydrate) under OA conditions at elevated temperature. While protein concentrations were either unaffected [30], [31] or increased in response to elevated *p*CO_2_ alone [13], [50], the effects of OA, or OA plus elevated temperature, on coral lipids and carbohydrates are unknown. Studies specifically addressing all three energy reserve pools are needed to get a better understanding of how OA affects coral energetics and their overall resistance to future climate change.

Finally, the algal endosymbiont (*Symbiodinium* sp.) provides healthy corals with up to 100% of their daily metabolic energy demand via photosynthesis [51]. If algal productivity is enhanced under OA due to CO_2_ fertilization [41], this might help maintain calcification rates and/or energy reserves under OA as energetic costs for calcification increase. Further, *Symbiodinium* sp. exhibit high sensitivity to elevated seawater temperature [52]. Thus, it is important to monitor endosymbiont and chlorophyll *a* concentrations in studies manipulating both *p*CO_2_ and temperature.

Here, we studied the single and interactive effects of *p*CO_2_ (382, 607, 741 µatm) and temperature (26.5 and 29.0°C) on coral calcification, energy reserves (i.e., lipid, protein, and carbohydrate), chlorophyll *a*, and endosymbiont concentrations in 4 species of Pacific coral with different growth morphologies. It was hypothesized that 1) calcification and energy reserves decrease in response to elevated *p*CO_2_ and elevated temperature, 2) decreases are larger when *p*CO_2_ and temperature are elevated simultaneously, and 3) that physiological responses are species-specific. We show that only one of the four coral species studied here decreased calcification in response to average ocean acidification levels expected by the second half of this century (741 µatm), even when combined with elevated temperature (+2.5°C). Further, we show for the first time that energy reserves were largely not metabolized in order to sustain calcification under elevated *p*CO_2_ and temperature, suggesting that some coral species will be more resistant to combined ocean acidification and warming than previously expected.

## Materials and Methods

### Experiment

Six parent colonies of *Acropora millepora, Pocillopora damicornis, Montipora monasteriata,* and *Turbinaria reniformis* were purchased from Reef Systems Coral Farm (New Albany, Ohio, USA) which is a CITES permit holder. The parent colonies were specifically collected for this experiment from 3–10 m in northwest Fiji (17°29′19″S, 177°23′39″E) in April 2011. Colonies of the same species were collected at least 10 m apart to increase the probability that different genotypes of the same species were selected. All colonies were shipped to Reef Systems Coral Farm and maintained in recirculating indoor aquaria with natural light (greenhouse, 700–1000 µmol quanta m^−2^ s^−1^) and commercially available artificial seawater (Instant Ocean Reef Crystals) for 2.5 months until the start of the experiment.

**Figure 1:**
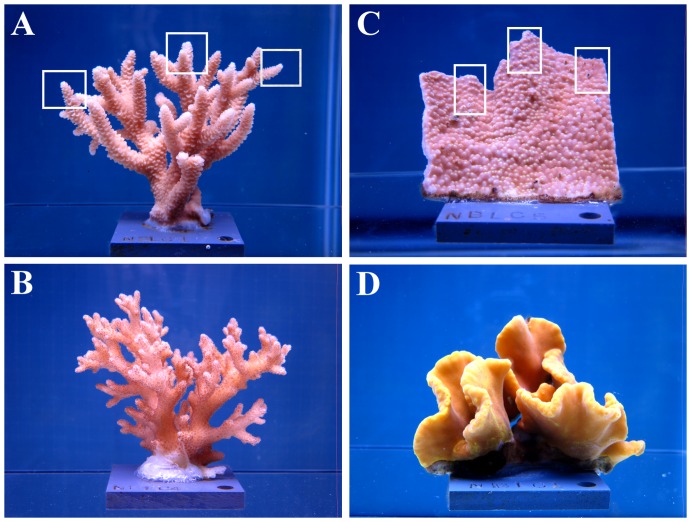
Photos of representative coral fragments from (a) Acropora millepora, (b) Pocillopora damicornis, (c) Montipora monasteriata, and (d) Turbinaria reniformis. Rectangles indicate subsamples taken from each fragment for lipid, protein/carbohydrate, and tissue biomass analyses. The remaining tissue was airbrushed for chlorophyll *a* and endosymbiont density measurements.

From April 22 - May 19, 2011, six fragments were collected from each parent colony and mounted on PVC tiles for a total of 144 fragments (4 species × 6 colonies × 6 fragments; Fig. 1). Starting on June 19, 2011, corals were gradually acclimated to a custom-made artificial seawater (ESV Aquarium Products Inc.), which was designed to mimic the chemical composition and alkalinity of natural reef seawater. On July 8 and 9, 2011, all 144 fragments were transferred to the experimental recirculating indoor aquaria with artificial light (Tek Light T5 actinic lights, 275 µmol quanta m^−2^ s^−1^, 9∶15 hrs light:dark cycle) and allowed to acclimate to the artificial light conditions for 10 days under ambient seawater conditions (i.e., 26.5°C and *p*CO_2_ of 382 µatm). Photosynthesis to irradiance (P/E) curves performed on *Acropora millepora* showed that photosynthesis was fully saturated at these light levels. Due to logistical reasons, P/E curves were not performed on the other species.

For each of the 6 treatments, the recirculating tank system consisted of one 905 L sump and six aquaria of 57 L each. One fragment per parent colony per species was put in one of the 6 aquaria in each system such that there were a total of 4 fragments (one of each species) in each aquarium, and each parent colony of each species was represented in each system. By placing the same genotypes in each treatment, genotypic variation between treatments was minimized and our ability to detect treatment effects was optimized. Replication of treatments and independent tanks within treatments was not possible due to the complexity and cost of operating tanks under modified pH conditions. While this is, strictly speaking, a pseudo-replicated design [53], the disadvantages of this design are outweighed by the advantages of being able to simultaneously manipulate six combinations of temperature and pH. To optimize the experimental design conditions, coral fragments were rotated daily within tanks and every 3 days among tanks within each system to minimize any tank or positional effects within each system. Further, tanks were cleaned every three days, and great care was taken to ensure similar conditions across treatments except for carbonate chemistry and temperature.

Experimental treatments were assigned to each system as follows: 26.5°C and 382 µatm, 26.5°C and 607 µatm, 26.5°C and 741 µatm, 29.0°C and 382 µatm, 29.0°C and 607 µatm, and 29.0°C and 741 µatm (Fig 1). The three *p*CO_2_ levels-382, 607, and 741 µatm – were designed to represent present day *p*CO_2_, and two *p*CO_2_ levels expected by the second half of the 21^st^ century, respectively. The control temperature (26.5°C) represents the current average annual temperatures in Fiji (http://www.ospo.noaa.gov/Products/ocean/index.html), whereas 29.0°C represents the upper limit of current summer temperatures but is still below the bleaching threshold at that location. Therefore, the 26.5°C and 382 µatm treatment served as control. The experiment lasted for 24 days from July 19-August 12, 2011.

Temperature was controlled by titanium aquarium heaters submerged in each system sump (Aqua Medic) and connected to a digital control system (Neptune Systems Apex AquaController). Temperature loggers (Onset Hobo Pro v2) were placed in each sump and recorded temperature every 5 minutes. Seawater *p*CO_2_ was controlled by bubbling in pure CO_2_, CO_2_-free air, or ambient air delivered by an outdoor air pump (Sweetwater, Aquatic Eco-Systems Inc.) into each system sump. CO_2_-free air was achieved by moving ambient air through CO_2_-scrubbers consisting of a 1.5 m long tube (10 cm diameter) filled with soda lime (SodaSorb HP). Supply of all gases was controlled via a pH stat system using custom designed software (KSgrowstat, written by K. Oxborough, University of Essex). Seawater pH was measured every 5 seconds by microelectrodes (Thermo Scientific Orion Ross Ultra pH glass electrode), which were calibrated daily.

For the elevated temperature (29.0°C) treatments, temperature was gradually increased over several days until the desired temperature was reached. For the medium (607 µatm) and high (741 µatm) *p*CO_2_ treatments, *p*CO_2_ was gradually increased over several days starting from 382 µatm until the final *p*CO_2_ was achieved. Recirculating seawater flow rate was 210–230 l/hour and little pumps (Accela Powerheads) created additional water circulation within each aquarium. A quarter of the entire water volume of each treatment system was exchanged every 3 days. Non-carbonate ceramic filter media (MarinePure High Performance Biofilter Media, CerMedia) were placed in the sumps to filter the water. Tanks were cleaned every 3 days or as needed.

Since healthy corals *in situ* can acquire up to 46% of their daily metabolic energy demands by feeding on zooplankton [48], [54], corals were fed every three days with 48 h old brine shrimp nauplii (*Artemia* sp., Carolina Biological Supply). Corals were allowed to acclimate to the dark for 30 min before feeding was conducted. They were fed for one hour in separate, partially submerged plastic containers containing water from their respective treatment, and at a concentration of approximately 1 brine shrimp ml^−1^ which is representative of zooplankton concentrations on natural Pacific reefs [55]. At the end of the hour, brine shrimp nauplii remained in the feeding chambers indicating that the corals had not captured all brine shrimp nauplii available to them. Following feeding, the corals were placed back in their respective aquaria and the feeding container water discarded so as not to introduce brine shrimp into the recirculating systems.

### Monitoring of seawater chemistry during the experiment

Temperature and salinity were measured daily (YSI 63), and salinity was adjusted daily to 35 ppt. Daily water samples were taken using screw-top high-density polyethylene bottles for pH and alkalinity analyses. After equilibration at 25°C in a recirculating water bath (30 min),, sample pH_NBS_ was measured with an Orion® Ross glass electrode (precision 0.01 pH units) [56], which was calibrated daily at 25°C. Total alkalinity (TA) was titrated with HCl on the same samples using an AS-ALK2 (Apollo SciTech Inc.) alkalinity titrator [57] (precision 0.1%). The HCl solution was calibrated with Certified Reference Material (CRM) from A.G. Dickson (Scripps).

Treatment xCO_2_ (dry air), aragonite saturation state (Ω_arag_), and pH_T_ were calculated using the program CO2SYS [58] based on measured pH_NBS_ and alkalinity at the respective temperature. xCO_2_ was converted to *p*CO_2_ using the equation in Weiss *et al*. [59]. Carbonate dissociation constants were taken from Millero *et al.* [60]. In addition, a custom-made CO_2_ analyzer based on a LI-COR 820 was used weekly to crosscheck with calculated sump *x*CO_2_ values according to methods by Wang & Cai [56], and indicated good agreement of measured and calculated values (*r* ^2^ = 0.97, n = 66).

### Laboratory analyses

#### Calcification

Net calcification was determined using the buoyant weight technique [61]. Each coral fragment was buoyantly weighed at the beginning, middle (after 11 experimental days), and at the end of the experiment (after 23 experimental days). As such, it was possible to assess if calcification rates varied during the experiment. Daily calcification rates were calculated as the difference between initial, middle, and final weights, divided by the respective number of days elapsed, and standardized to surface area (see below).

For tissue analyses, corals were frozen at −80°C and a total of three branch tips or growing edge pieces were saved from each fragment for lipid, protein/carbohydrate, and tissue biomass analyses, respectively (Fig. 1). The remaining tissue was airbrushed for chlorophyll *a* and endosymbiont density measurements.

#### Chlorophyll a and endosymbiont density

Coral tissue was stripped off the coral skeleton with a waterpik [62] containing 40 ml of synthetic seawater (Instant Ocean). The endosymbionts were isolated from the host tissue via centrifugation and then resuspended in 10 ml of synthetic seawater. For chlorophyll *a* concentrations, 1 ml of this algal suspension was pelleted and the cells lysed in 1 ml of 4°C methanol using a bead-beater for 60 seconds. Samples were then immediately placed on ice and allowed to extract for one hour in the dark. Samples were centrifuged to remove cellular debris and measured spectrophotometrically (λ = 652, 665 & 750) on a 96-well plate reader. The equations for chlorophyll *a* in methanol described by Porra *et al.* [63], along with path length correction [64], were used to calculate chlorophyll *a* concentrations (pg/cell), and were then standardized to surface area (see below). Another 1 ml subsample of the algal suspension was preserved with 10 µl of 1% glutaraldehyde solution for endosymbiont quantification, which was calculated using 6 independent replicate counts on a hemocytometer, using a Nikon microphot-FXA epifluorescent microscope at 100× magnification. Photographs were analyzed through Image J using the analyze particles function.

#### Energy reserves and tissue biomass

For all energy reserve and tissue biomass measurements, only branch tips or samples with a growing edge were used. While tissue composition may vary across the surface of a coral [65], this approach was used to allow for comparison with previously published studies [46], [47], [66]. Soluble lipids (referred to hereafter simply as lipids) were extracted from a whole, ground coral sample (skeleton + animal tissue + algal endosymbiont) in a 2∶1 chloroform:methanol solution for 1 hour [46], [66] washed in 0.88% KCl followed by 100% chloroform and another wash with 0.88% KCl. The extract was dried to constant weight under a stream of pure nitrogen (UPH grade 5.0) and standardized to the ash-free dry weight.

Animal soluble protein and carbohydrate (referred to hereafter simply as protein and carbohydrate, respectively) were extracted from grinding a whole second branch tip of the same fragment [46]. Briefly, Milli-Q water was added to the ground coral sample and the resulting slurry was sonicated (5 min) and then centrifuged twice (5000 rpm, 10 min) to separate the animal tissue from the skeleton and endosymbiotic algae. Protein and carbohydrate was extracted from the animal tissue only. One subsample of this animal tissue slurry was used for protein extraction using the bicinchoninic acid method [67] with bovine serum albumin as a standard (Pierce BCA Protein Assay Kit). A second subsample was used for carbohydrate quantification using the phenol-sulfuric acid method [68] with glucose as a standard. Soluble animal protein and carbohydrate concentrations were standardized to the ash-free dry weight.

Tissue biomass was measured by drying a third branch tip of whole coral sample (skeleton + animal tissue + algal endosymbiont) to constant dry weight (24 hours, 60°C) and burning it (6 hours, 450°C). The difference between dry and burned weight was the ash free dry weight which was standardized to the surface area of this branch tip.

#### Surface area

Surface area of plating *M. monasteriata* and *T. reniformis* fragments was determined using the aluminum foil technique [69], whereas surface area of branching *A. millepora* and *P. damicornis* fragments was determined using the single wax dipping technique [70], [71] after the tissue had been removed. Natural wooden blocks of varying sizes and shapes were used as calibration standards [71]. Wax dipping was conducted using household paraffin wax (Gulf Wax, Royal Oak Enterprises) heated to 65°C. Dried coral skeletons and wooden calibration standards were maintained at room temperature prior to weighing.

### Statistical analyses

Three-way mixed-model analyses of variance (ANOVA) tested the effects of *p*CO_2_, temperature, and parent colony on calcification rates in the first and second half of the experiment, chlorophyll *a*, algal endosymbiont density, lipid, protein, carbohydrate, and tissue biomass. Temperature and *p*CO_2_ were fixed and fully crossed, whereas parent colony was a random factor. The ANOVAs were run for each species separately. All data were normally distributed according to plots of residuals versus predicated values for each variable, or transformed to meet the condition of normality. Outlier values greater than 3 times the interquartile range were excluded. Post hoc Tukey tests were performed when main effects were significant (*p*≤0.05). A posteriori slice tests (e.g., tests of simple effects) [72] determined if the ambient (26.5°C) and elevated (29.0°C) temperature treatment averages significantly differed within each *p*CO_2_ level. Bonferroni corrections were not applied [73], [74], therefore significant model *p*-values >0.0016 (0.05/32 tests) should be interpreted with caution. Statistical analyses were performed using SAS software, Version 9.2 of the SAS System for Windows.

## Results

All corals appeared healthy throughout the experiment. No visible paling and no mortality occurred. The average seawater temperature, pH_T_, *p*CO_2_, saturation state, and total alkalinity for all six treatments are summarized in Table 1.

**Table 1:** Average conditions for each of the 6 treatments representing three pCO_2_ levels at two temperature regimes (ambient, elevated  =  ambient + 2.5°C).

	400 ppm target		600 ppm target		800 ppm target	
	ambient temp.	elevated temp.	ambient temp.	elevated temp.	ambient temp.	elevated temp.
**Temp.(°C)**	26.45±0.01	29.31±0.02	26.37±0.01	28.53±0.02	26.61±0.01	28.93±0.02
						
**pH_T_**	8.07±0.01	8.04±0.01	7.90±0.01	7.89±0.01	7.83±0.01	7.81±0.01
						
***p*** **CO_2_ (ìatm)**	364.31±9.69	400.62±16.83	598.37±18.50	616.08±24.24	732.04±22.37	749.63±26.21
						
**TA (µmol kg^−1^)**	2269.4±10.84	2270.1±11.15	2303.8±9.34	2288.3±10.43	2306.3±10.64	2304.5±9.08
						
**Ω_arag_**	3.69±0.07	3.79±0.09	2.75±0.05	2.91±0.05	2.40±0.06	2.52±0.06

Mean ± 1 SE are shown. Sample size was 25 for all measurements. Temp.  =  Temperature.

### Calcification

**Figure 2:**
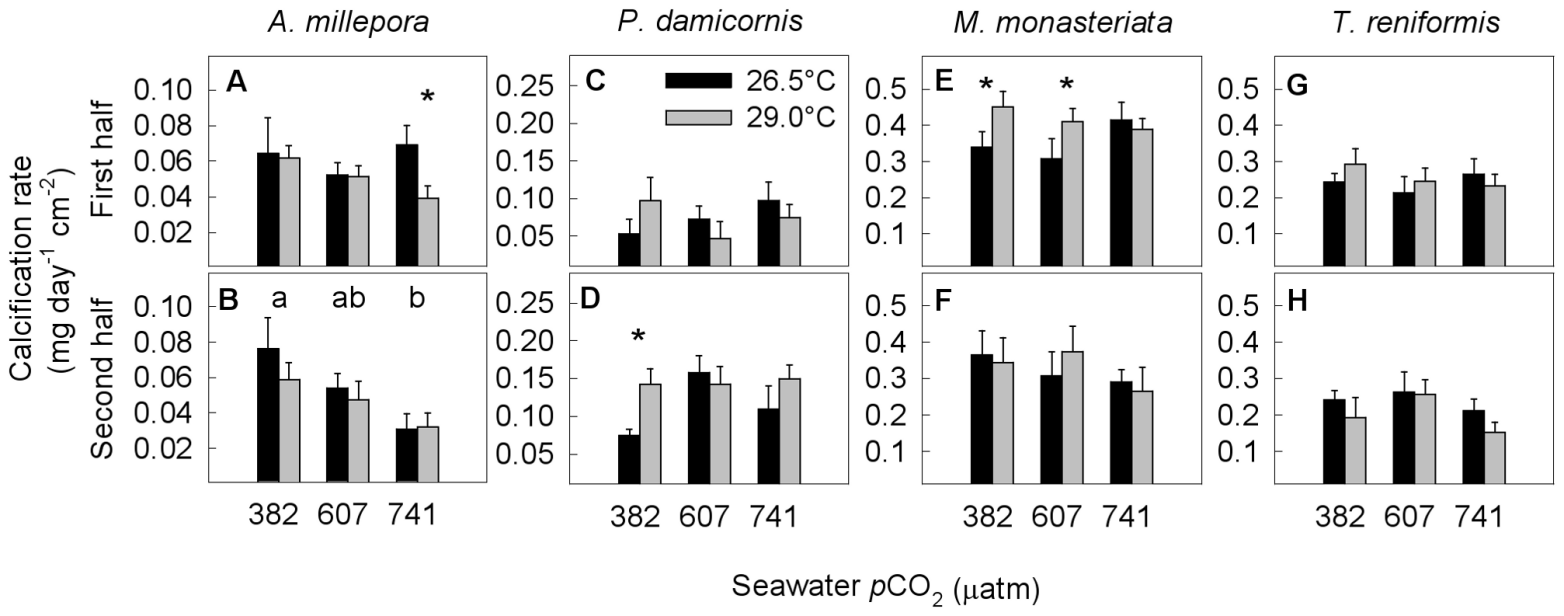
Average daily calcification rate during the first and the second half of the experiment for (a, b) Acropora millepora, (c, d) Pocillopora damicornis, (e, f) Montipora monasteriata, and (g, h) Turbinaria reniformis. Averages ± 1 SE are shown for three *p*CO_2_ levels and two temperature regimes (26.5, 29.0°C). Asterisks indicate significant differences between 26.5 and 29.0°C within a given *p*CO_2_ level (determined by a posteriori slice tests). The letters a and b indicate results of the post hoc Tukey tests when there was a significant *p*CO_2_ effect. Sample sizes ranged between 5 and 6. Statistical details can be found in Table S1.

In *Acropora millepora*, calcification rates during the first half of the experiment were overall unaffected by both temperature (*p* = 0.36) and *p*CO_2_ (*p* = 0.79) (Fig. 2a; Table S1). However, at the highest *p*CO_2_ level (741 µatm) calcification was 43% lower at 29.0°C than at 26.5°C (Fig. 2a). During the second half of the experiment, calcification rates were significantly affected by *p*CO_2_ (*p* = 0.001) but not temperature (*p* = 0.42), and were lower by 53% at the highest compared to the lowest *p*CO_2_ level (Fig. 2b; Table S1).

In *Pocillopora damicornis*, a significant interaction of *p*CO_2_ and temperature (*p*<0.001) was observed for calcification rates during the first half of the experiment (Fig. 2c, Table S1). During the second half of the experiment, calcification rates were generally unaffected by temperature (*p* = 0.06) and *p*CO_2_ (*p* = 0.07). However, at ambient seawater *p*CO_2_ (382 µatm) corals kept at elevated temperature (29.0°C) calcified 91% more compared to those kept at 26.5°C (Fig. 2d, Table S1).

Calcification rates of *Montipora monasteriata* were affected by temperature (*p* = 0.04) but not *p*CO_2_ (p = 0.42) during the first half of the experiment (Fig. 2e–f, Table S1), with corals calcifying 18% more at elevated compared to ambient temperature. This was largely driven by significant temperature differences at both 382 and 607 µatm but not 741 µatm. During the second half of the experiment, calcification rates were unaffected by both temperature (*p* = 0.82) and *p*CO_2_ (*p* = 0.14).

In contrast, calcification rates of *Turbinaria reniformis* during both first and second half of the experiment (Fig. 2g–h, Table S1) did not respond to changes in seawater temperature (*p* = 0.45 and 0.17) or *p*CO_2_ (*p* = 0.36 and 0.09). Notably, the two plating species (*M. monasteriata* and *T. reniformis*) calcified more than twice as fast as the two branching species (*A. millepora* and *P. damicornis*).

### Chlorophyll a and endosymbiont density

**Figure 3:**
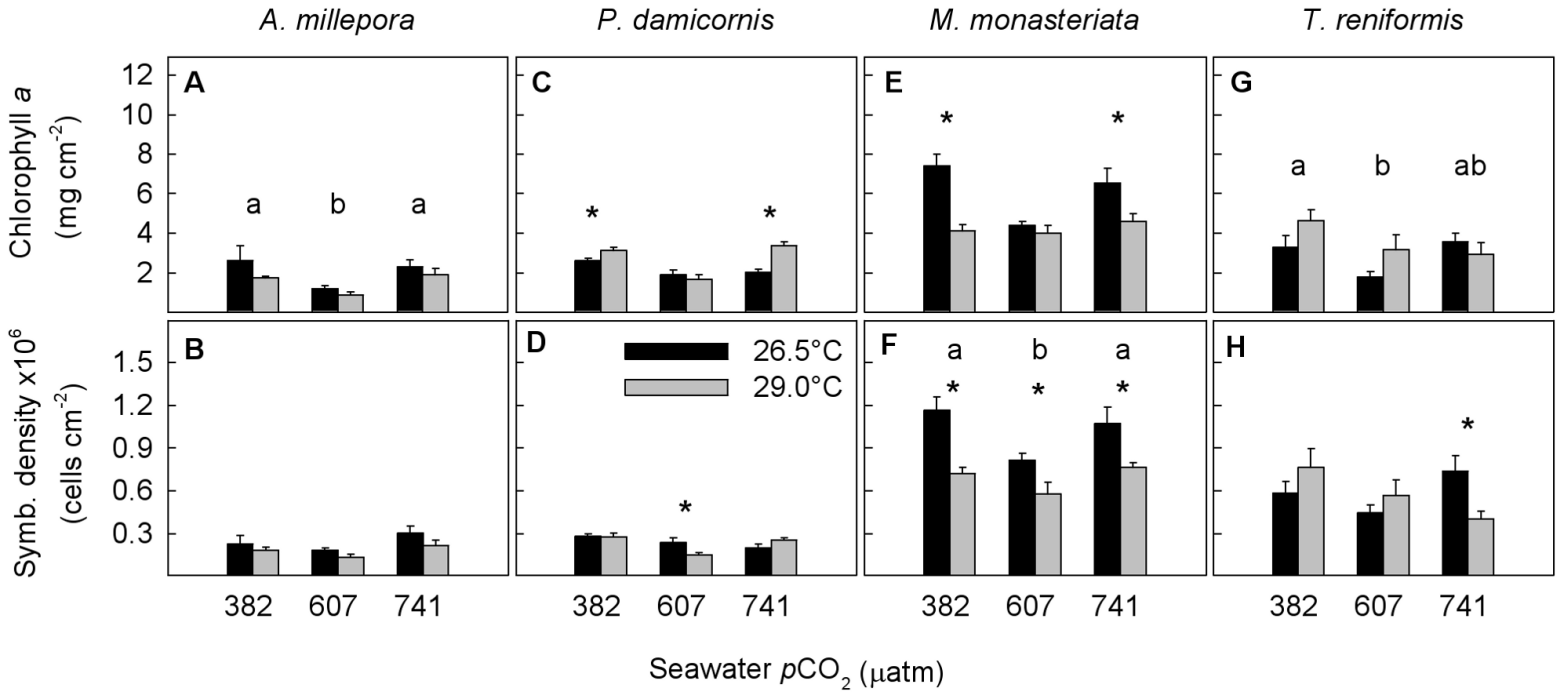
Average chlorophyll a concentrations and symbiont density for (a, b) Acropora millepora, (c, d) Pocillopora damicornis, (e, f) Montipora monasteriata, and (g, h) Turbinaria reniformis. Averages ± 1 SE are shown for three *p*CO_2_ levels and two temperature regimes (26.5, 29.0°C). Asterisks indicate significant differences between 26.5 and 29.0°C within a specific *p*CO_2_ level (determined by a posteriori slice tests). The letters a and b indicate results of the post hoc Tukey tests when there was a significant *p*CO_2_ effect. Sample sizes ranged between 5 and 6. Statistical details can be found in Table S2.

The chlorophyll *a* concentrations of *A. millepora* were significantly affected by *p*CO_2_ (*p*<0.001) but not temperature (*p* = 0.054), with concentrations being 51% lower at 607 µatm than at either 382 or 741 µatm (Fig. 3a, Table S2). Endosymbiont densities were not affected by either seawater temperature (*p* = 0.07) or *p*CO_2_ (*p* = 0.03 but overall model *p* = 0.24) (Fig. 3b, Table S2).

In *P. damicornis*, a significant interaction of temperature and *p*CO_2_ was observed for both chlorophyll *a* concentrations and endosymbiont densities (*p*<0.001 and *p* = 0.02, respectively) (Fig. 3c–d, Table S2). When temperature was elevated, chlorophyll *a* concentrations were higher by 19% and 67%, respectively, at both 382 and 741 µatm. At 607 µatm, endosymbiont densities decreased by 36% at 29°C compared to concentrations at ambient temperature.

In *M. monasteriata*, a significant interaction of seawater temperature and *p*CO_2_ was observed for chlorophyll *a* concentrations (*p* = 0.01) (Fig. 3e, Table S2), with concentrations being 45% and 30% lower at elevated compared to ambient temperature, respectively, under both 382 and 741 µatm conditions. Endosymbiont densities were significantly affected by both temperature (*p*<0.001) and *p*CO_2_ (*p* = 0.01) but the interaction term was not significant (*p* = 0.38) (Fig. 3f, Table S2). Densities were 32% lower at elevated compared to ambient temperature, and were lowest overall at 607 µatm (−25%) compared to the other two *p*CO_2_ levels.

Chlorophyll *a* concentrations of *T. reniformis* were affected by *p*CO_2_ (*p* = 0.03) but not temperature (*p* = 0.11), with concentrations being 38% lower at 607 compared to 382 µatm (Fig. 3g, Table S2). Endosymbiont densities were not affected by either temperature (*p* = 0.90) or *p*CO_2_ (*p* = 0.21) but were lower by 45% at elevated compared to ambient temperature under 741 µatm conditions (Fig. 3h, Table S2).

### Energy reserves and tissue biomass

**Figure 4:**
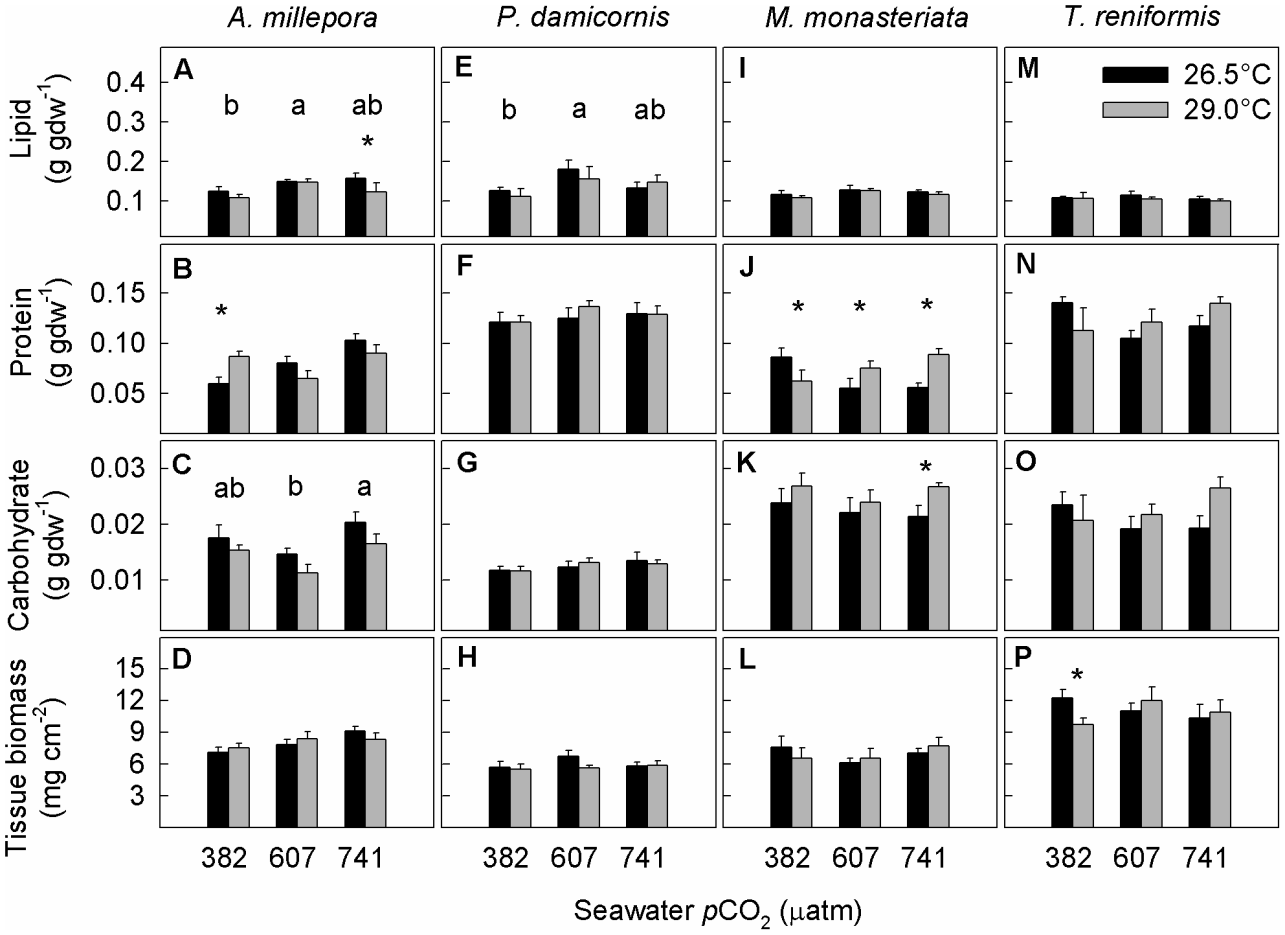
Average lipid, protein, carbohydrate concentrations, and tissue biomass of (a–d) Acropora millepora, (e–h) Pocillopora damicornis, (i–l) Montipora monasteriata, and (m–p) Turbinaria reniformis. Averages ± 1 SE are shown for three *p*CO_2_ levels and two temperature regimes (26.5, 29.0°C). Asterisks indicate significant differences between 26.5 and 29.0°C within a specific *p*CO_2_ level (determined by a posteriori slice tests). The letters a and b indicate results of the post hoc Tukey tests when there was a significant *p*CO_2_ effect. Sample sizes ranged between 4 and 6. Statistical details can be found in Table S3.

Lipid concentrations of *A. millepora* were affected by seawater *p*CO_2_ (*p* = 0.01) but not temperature (*p* = 0.053), with concentrations being 28% and 21% higher at 607 and 741 µatm, respectively, compared to concentrations at ambient *p*CO_2_ (Fig. 4a, Table S3). A significant interaction of seawater *p*CO_2_ and temperature was observed for protein concentrations (*p* = 0.01) (Fig. 4b, Table S3). Carbohydrate concentrations were affected by both temperature (*p* = 0.02) and *p*CO_2_ (*p* = 0.01) but the interaction term was not significant (*p* = 0.85) (Fig. 4c, Table S3). Across all *p*CO_2_ treatments, carbohydrate concentrations were 18% lower at 29.0°C compared to 26.5°C, and 41% lower at 607 µatm than at 741 µatm. Tissue biomass was unaffected by changes in seawater temperature (*p* = 0.99) and *p*CO_2_ (*p* = 0.07) (Fig. 4d, Table S3).

In *P. damicornis*, lipid concentrations were affected by seawater *p*CO_2_ (*p* = 0.01) but not temperature (*p* = 0.53) (Fig. 4e, Table S3), with concentrations being 41% and 18% higher at 607 and 741 µatm, respectively, compared to concentrations at ambient *p*CO_2_ (Fig. 4e, Table S3). Neither protein, nor carbohydrate concentrations or tissue biomass were affected by seawater temperature (*p* = 0.63, 0.88, 0.33, respectively) and *p*CO_2_ (*p* = 0.52, 0.35, 0.41, respectively) (Fig. 4f–h, Table S3).

The lipid concentrations of *M. monasteriata* were unaffected by both seawater temperature (*p* = 0.38) and *p*CO_2_ (*p* = 0.23) (Fig. 4i, Table S3). A significant interaction of temperature and *p*CO_2_ was observed for protein concentrations (*p*<0.001): at 382 µatm, they decreased (−27%) at elevated compared to ambient temperature, whereas at 607 and741 µatm, they increased (+36% and +60%, respectively) (Fig. 4j, Table S3). Carbohydrate concentrations were affected by seawater temperature (*p* = 0.02) but not *p*CO_2_ (*p* = 0.36) (Fig. 4k, Table S3), and the concentrations were 25% higher at elevated than at ambient temperature under 741 µatm conditions. Tissue biomass was also unaffected by both seawater temperature (*p* = 0.78) and *p*CO_2_ (*p* = 0.12) (Fig. 4l, Table S3).

In *T. reniformis*, none of the measured energy reserve pools responded to changes in seawater temperature and *p*CO_2_ (Fig. 4m–o, Table S3). Tissue biomass was also unaffected by both temperature (*p* = 0.62) and *p*CO_2_ (*p* = 0.58), but was 21% lower at 29.0°C compared to 26.5°C at 382 µatm (Fig. 4p, Table S3).

### Effects of parent colony

Parent colony was a significant effect in many of the measured variables, but no single parent colony or group of specific parent colonies was consistently different from all other parent colonies in any of the species studied (Tables S1–S3).

## Discussion

Coral calcification has been predicted to decrease dramatically by the end of this century, thus threatening the existence of coral reefs in the future. Although the response of coral calcification is not uniform across species, most studies have found that calcification decreases with increasing seawater *p*CO_2_ [75]–[77]. Here, we show that only one of the four Pacific coral species studied here decreased calcification in response to average ocean acidification levels expected by the second half of this century (741 µatm), even when combined with elevated temperature (+2.5°C). Further, we investigated for the first time the effects of OA on coral energy reserves and show that they were largely not metabolized in order to sustain calcification under elevated *p*CO_2_ and temperature.

*Acropora millepora* was the only coral out of the four species studied here that decreased calcification rates in response to OA (Fig. 2b). While calcification rates were not affected by elevated *p*CO_2_ and/or temperature during the first half of the experiment, they declined by 53% during the second half of the experiment due to acidification alone. As the second half of the experiment is more likely to reflect the long term response of corals to ocean acidification, this negative response to OA is consistent with other studies on *Acropora* sp. [14], [20], [24], [78], although the amount of decline differs between species. The absence of any change in calcification of *Pocillopora damicornis* is consistent with another study [14], whereas declines in calcification of 50% in *P. meandrina* were reported [37]. The lack of any change in calcification rates of *Montipora monasteriata*, and *Turbinaria reniformis* due to acidification (Fig. 2d, f, h) is in contrast to other studies which reported a 15–20% decline in *Montipora capitata* [16], and a 13% decline in *T. reniformis* (albeit at *p*CO_2_ levels that were considerably higher than those in the present study) [78]. Although a significant interaction of *p*CO_2_ and temperature was observed in *P. damicornis* during the first half of the experiment (Fig. 2c), this was not observed during the second half. Similarly, *M. monasteriata* calcified more at elevated compared to ambient temperature during the first half (Fig. 2e), but not during the second half of the experiment. Thus, it appears that with the exception of *A. millepora*, these species may be resistant to changes in *p*CO_2_ and temperature within the parameter ranges investigated in this study.

In the current study, elevated temperature did not exacerbate or counteract the negative effects of OA on calcification in *A. millepora*, and did not have an overall negative affect on calcification in the other three species. This is in contrast to other studies where elevated temperature was found to mitigate negative OA effects. For example, Anthony *et al.* [20] found that elevated temperature (28–29°C vs. 25–26°C) prevented a decline of calcification in *A. intermedia* at elevated *p*CO_2_ (520–705 µatm). Muehllehner and Edmunds [37] showed that the negative effects of elevated *p*CO_2_ (720 µatm) were fully mediated in *P. meandrina* when OA was combined with elevated temperature (29°C vs. 27°C). Overall, these findings add to the growing body of evidence that the response of coral calcification to OA is highly species specific, and that some coral species may maintain calcification under combined ocean acidification and warming in the future.

Although the current study was conducted using artificial seawater, it is unlikely that this influenced the observed responses of calcification to ocean acidification. The carbonate chemistry of the custom-made seawater mimicked natural conditions very well (Table 1), and calcification rates – as well as chlorophyll *a* concentrations, endosymbiont densities, energy reserves, and tissue biomass – were within the range observed in the field and/or other experimental studies using natural seawater [13], [28], [43], [45], [46], [66], [78]–[89].

While many studies note a decline in coral calcification with increasing *p*CO_2_ [75]–[77], there is considerable among-study variation [75], [76], and some species are more resistant than others [15], [90]. Such differences may be due to experimental duration, how seawater carbonate chemistry is altered (i.e., bubbling CO_2_ vs. acid addition), and how calcification is measured (i.e., buoyant weight vs. total alkalinity anomaly technique). Meta-analyses have shown that experimental duration or the method of carbonate chemistry manipulation did not explain the large variability of responses observed among studies [75], [76]. While this study suggests that experimental duration can influence the response of calcification to OA in some species (i.e., calcification of *A. millepora* decreased only during the second half of the second half), it is likely that biological aspects have a stronger influence on the sensitivity of coral calcification to OA than differences in methodology. Important biological aspects include energetic status and feeding [28], [38], enhanced algal production [41], [91], and cellular pH control [92]–[95].

Despite the assumption that calcification becomes energetically more costly under OA [38]–[40], energy reserves did not decline with increasing *p*CO_2_ (Fig. 4). Lipid concentrations increased under OA conditions in both *A. millepora* and *P. damicornis*, and were fully maintained in *M. monasteriata* and *T. reniformis*. Protein, carbohydrate, and tissue biomass were overall maintained under OA conditions in all species. Further, temperature did not negatively affect energy reserves and tissue biomass except for carbohydrate concentrations in *A. millepora*, which were lower at elevated compared to ambient temperature. Importantly, energy reserves and tissue biomass were fully maintained or even increased at the highest *p*CO_2_ level in *A. millepora* despite dramatic decreases in calcification rates. These findings suggest that (1) energy reserves are generally not metabolized under OA conditions or OA at elevated temperature, and (2) that either energy reserves do not play a role in sustaining calcification under OA conditions, or that the increased energetic costs of maintaining calcification under OA are relatively insignificant. This is consistent with other work showing that calcification likely does not become energetically more costly under OA conditions [81], and that the extra energy required to up-regulate pH at the site of calcification under OA conditions is <1% of that produced by photosynthesis [92].

Further, from an energetic standpoint of view, the total amount of energy reserves present in a coral species did not seem to be related to their calcification response to OA. The energetic content of lipid, protein, and carbohydrates is better assessed from an energetic point of view [96], [97], as specific enthalpies of combustion differ significantly among energy reserve pools: -39.5 kJ g^−1^ for lipid, −23.9 kJ g^−1^ for protein, and −17.5 kJ g^−1^ for carbohydrate [96]. When the total amount of energy available to each species was calculated (i.e., the sum of lipid, protein, and carbohydrate expressed in kJ g^−1^ tissue biomass), *A. millepora* had the lowest amount of all species in the control treatment (6.6 vs. up to 8.1 kJ g^−1^ in *P. damicornis*), but the highest amount in the high-CO_2_ treatment (9.0 vs. 6.6 kJ g^−1^ in *M. monasteriata*), and a similar amount as both *M. monasteriata* and *T. reniformis* in the high-CO_2_ – high temperature treatment (7.3 kJ g^−1^ vs. 7.2 and 7.8 kJ g^−1^, respectively). It is therefore unlikely that high levels of energy reserves *per se* help corals maintain calcification rates under OA conditions.

However, maintaining energy reserves and tissue biomass under ocean acidification does have crucial implications for other aspects of coral health and resistance to stressors such as coral bleaching. For example, maintenance of lipid concentrations may enable corals to maintain their reproductive output [98], even under future OA and warming. This may be critical considering that many other processes involved in coral reproduction such as fertilization, settlement success, and metamorphosis are compromised under OA [99], [100]. Furthermore, maintenance of energy reserves has been shown to be associated with higher resistance to coral bleaching and to promote recovery from bleaching [46], [49], which could prove critical as bleaching events will increase in frequency over the coming decades [101].

Heterotrophy is known to promote energy storage, tissue synthesis, and skeletal growth in healthy and bleached corals [47], [48], [102] as well as corals subjected to OA [28], [42]. Therefore, biweekly feeding in this study (intended to mimic zooplankton contribution to the coral diet on the reef) may have helped corals to sustain energy reserves and tissue biomass under these conditions. It has further been suggested that coral tissue reacts to availability of such resources faster than skeletal growth [103], [104], which could explain why tissue biomass - but not necessarily calcification – was maintained or even increased in all four species irrespective of *p*CO_2_ or temperature conditions. As feeding rates and heterotrophic plasticity are highly species-specific [48], [54], [105], it is likely that heterotrophic carbon intake differed significantly among the species studied here, potentially contributing to their differential responses to OA.

Enhanced algal productivity due to CO_2_-fertilization [41], [91] may help corals to maintain calcification under OA conditions. Although chlorophyll *a* concentrations and endosymbiont density were unaffected at the highest *p*CO_2_ level (except for chlorophyll in *T. reniformis*), CO_2_-fertilization may nevertheless have played a role in helping corals to maintain energy reserves and/or calcification. Increased availability of CO_2(aq)_ under OA conditions may enhance algal productivity, especially in *Symbiodinium* phylotypes with less efficient carbon-concentrating mechanisms, which rely to a greater extent on the passive, diffusive uptake of CO_2(aq)_ [41]. Thus, a potentially increased translocation of autotrophic carbon to the animal host may have contributed to the maintenance of energy reserves and tissue biomass observed here.

Interestingly, both chlorophyll *a* concentrations and endosymbiont density were often lowest at 607 µatm, showing a non-linear relationship with increasing *p*CO_2_. Nevertheless, the lack of any significant difference in chlorophyll *a* and/or symbiont density at 741 µatm versus ambient *p*CO_2_ concentrations (except for chlorophyll in *T. reniformis*) is consistent with other studies [23], [28], [29], [106]. The reason for the observed minima at ∼600 µatm is unknown. Similar non-linear responses were not observed for calcification rates, tissue biomass, and most energy reserve pools, suggesting that this did not translate into a decreased performance of the animal host. Edmunds [81] also observed a non-linear *p*CO_2_ threshold between 756 and 861 µatm affecting photochemistry and respiration in massive *Porites* corals, thus highlighting the importance of studying multiple *p*CO_2_ levels in OA experiments in order to assess non-linear physiological responses and to better forecast physiological responses over the coming century as the oceans continue to warm and acidify.

In addition to energetic status and enhanced algal productivity due to CO_2_ fertilization, other factors such as the amount of control over the carbonate chemistry at the site of calcification may explain the observed differences in susceptibility of calcification to OA. Corals have the ability to significantly up-regulate the pH at the site of calcification compared to ambient seawater, even under OA conditions [92]–[95], [107], [108]. Yet, the degree to which corals are able to control the pH at the site of calcification likely varies among species [92], [95], [109]. *Acropora* spp. may have the lowest capacity to up-regulate pH at the site of calcification based on boron isotopic measurements [92], [110]. Further, crystallization under OA was most compromised in *Acropora verweyi* and least compromised in *T. reniformis* [78]. Thus, we hypothesize that *A. millepora* has a weaker proton pump than the other coral species studied here, making its calcification rate more sensitive to future OA. Although pH up-regulation has not been studied in *P. damicornis, M. monasteriata*, or *T. reniformis*, it can be hypothesized that they have stronger control over the pH at the site of calcification and were therefore able to maintain calcification under the *p*CO_2_ levels studied here.

As physiological responses of both the animal host and algal endosymbiont to combined OA and warming were strongly species-specific, a wide range of susceptibility patterns can be expected resulting in ecological “winners and losers” [90], [111]. Branching *Acropora* corals, which are important reef builders, are likely to be “losers” on future coral reefs because they are highly susceptible to both bleaching [111] and OA [20], [24], [90], [99]. This can be expected to have severe impacts on reef diversity, structural complexity, and overall reef functioning. Nevertheless, some corals could be more resistant to combined ocean acidification and warming expected by the end of this century than previously thought, as three of the four species fully maintained calcification under elevated *p*CO_2_ and temperature without compromising overall energy reserves or biomass. Further, the immediate effects of rising seawater temperature and ocean acidification may be tolerable for some species.

## Supporting Information

Table S1 Results of 8 two-way ANOVAs for average calcification rate during the first and second half of the experiment.
(DOCX)

Table S2 Results of 8 two-way ANOVAs for average chlorophyll a concentrations and symbiont density.
(DOCX)

Table S3 Results of 16 two-way ANOVAs for average soluble lipid, animal soluble protein, animal soluble carbohydrate concentrations, and tissue biomass.
(DOCX)
