# Correction: Coral Energy Reserves and Calcification in a High-CO_2_ World at Two Temperatures

**DOI:** 10.1371/journal.pone.0108082

**Published:** 2014-09-08

**Authors:** The PLOS ONE Staff

There is an error in Table 1; the unit for *p*CO_2_ should read “µatm”. In addition, there is an error in Figure 3; the unit for Chlorophyll *a* should read “µg/cm^-2^.” Please see the correct Table 1 and Figure 3 here.

**Table 1:** Average conditions for each of the 6 treatments representing three pCO2 levels at two temperature regimes (ambient, elevated  =  ambient + 2.5°C).

	400 ppm target		600 ppm target		800 ppm target	
	ambient temp.	elevated temp.	ambient temp.	elevated temp.	ambient temp.	elevated temp.
**Temp. (°C)**	26.45 ± 0.01	29.31 ± 0.02	26.37 ± 0.01	28.53 ± 0.02	26.61 ± 0.01	28.93 ± 0.02
**pH_T_**	8.07 ± 0.01	8.04 ± 0.01	7.90 ± 0.01	7.89 ± 0.01	7.83 ± 0.01	7.81 ± 0.01
***p*** **CO_2_ (µatm)**	364.31± 9.69	400.62 ± 16.83	598.37 ± 18.50	616.08 ± 24.24	732.04 ± 22.37	749.63 ± 26.21
**TA (µmol kg^-1^)**	2269.4 ± 10.84	2270.1 ± 11.15	2303.8 ± 9.34	2288.3 ± 10.43	2306.3 ± 10.64	2304.5 ± 9.08
**Ω_arag_**	3.69 ± 0.07	3.79 ± 0.09	2.75 ± 0.05	2.91 ± 0.05	2.40 ± 0.06	2.52 ± 0.06

Mean ± 1 SE are shown. Sample size was 25 for all measurements. Temp.  =  Temperature.

**Figure 3:**
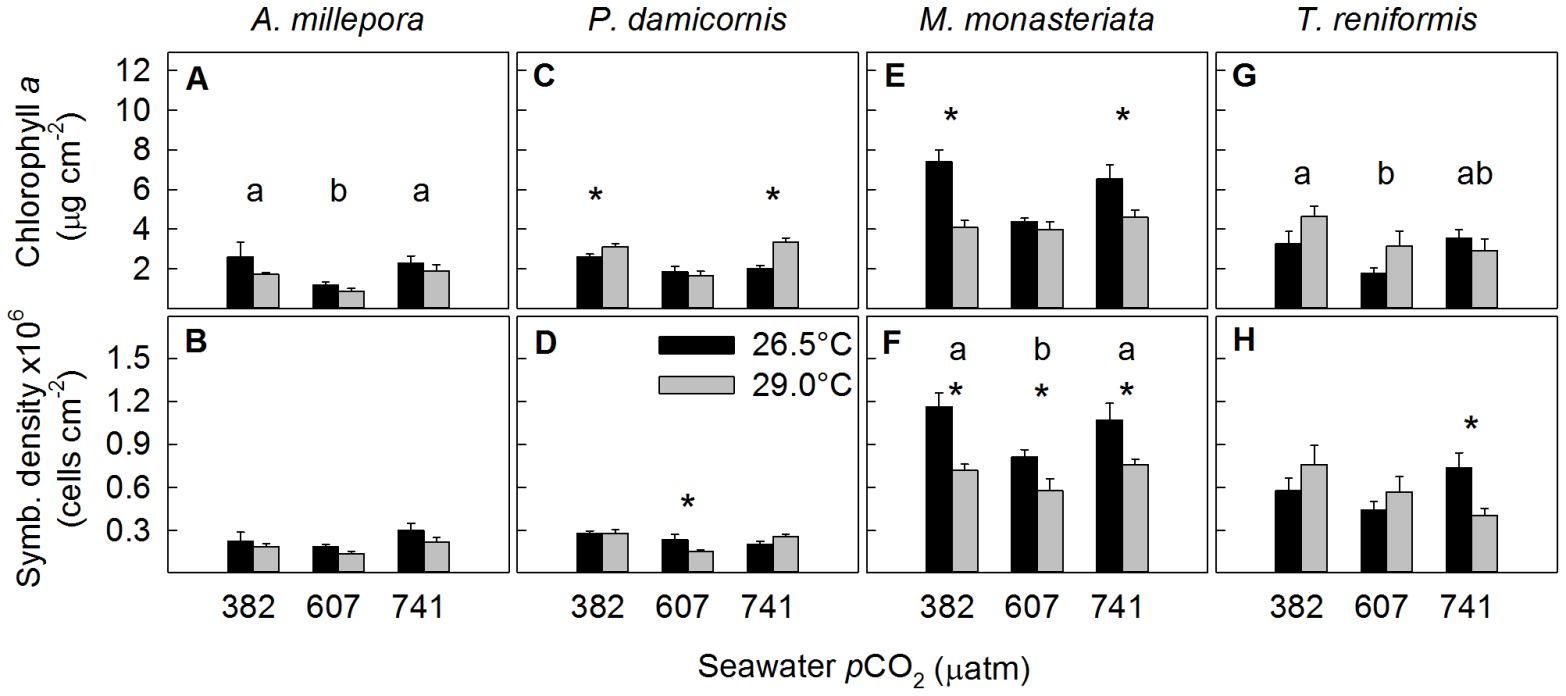
Average chlorophyll a concentrations and symbiont density for (a, b) Acropora millepora, (c, d) Pocillopora damicornis, (e, f) Montipora monasteriata, and (g, h) Turbinaria reniformis. Averages ± 1 SE are shown for three *p*CO_2_ levels and two temperature regimes (26.5, 29.0°C). Asterisks indicate significant differences between 26.5 and 29.0°C within a specific *p*CO_2_ level (determined by a posteriori slice tests). The letters a and b indicate results of the post hoc Tukey tests when there was a significant *p*CO_2_ effect. Sample sizes ranged between 5 and 6. Statistical details can be found in Table S2.
