# Supplementary material for: Coral Energy Reserves and Calcification in a High-CO2 World at Two Temperatures
Source: PLoS One. 2013 Oct 11;8(10):e75049. doi: 10.1371/journal.pone.0075049 (PMC3795744; doi:10.1371/journal.pone.0075049)
Supplement: Table S1 — Results of 8 two-way ANOVAs for average calcification rate during the first and second half of the experiment. (DOCX) [file pone.0075049.s001.docx]

**Coral energy reserves and calcification in a high-CO_2_ world at two temperatures**

Verena Schoepf^1^, Andréa G. Grottoli^1^, Mark E. Warner^2^, Wei-Jun Cai^3,*^, Todd F. Melman^4^, Kenneth D. Hoadley^2^, D. Tye Pettay^2^, Xinping Hu^3,†^, Qian Li^3,‡^, Hui Xu^3,∆^, Yongchen Wang^3^, Yohei Matsui^1^, Justin H. Baumann^1^

**Author affiliations**

1. School of Earth Sciences, The Ohio State University, Columbus, OH, United States
2. School of Marine Science and Policy, University of Delaware, Lewes, DE, United States
3. Department of Marine Sciences, University of Georgia, Athens, GA, United States
4. Reef Systems Coral Farm, New Albany, OH, United States

* Present address: School of Marine Science and Policy, University of Delaware, Newark, DE, United States

† Present address: Department of Physical and Environmental Sciences, Texas A&M University, Corpus Christi, TX, United States

‡ Present address: State Key Laboratory of Marine Environmental Science, Xiamen University, Xiamen, China

∆ Present address: Department of Ocean Science and Engineering, Zhejiang University, Hangzhou, China

**Corresponding author**

Verena Schoepf

E-mail: schoepf.4@osu.edu

**Supplemental Table S1. *Results of 8 two-way ANOVAs for average calcification rate during the first and second half of the experiment.*** Four species (*Acropora millepora, Pocillopora damicornis, Montipora monasteriata, Turbinaria reniformis*) were compared at three *p*CO_2_ concentrations (382, 607, 741 μatm) and two temperature levels (26.5, 29.0°C) with colony as a random factor. Post hoc Tukey tests were used when main effects were significant. Effects were considered significant when *p*≤0.05 (highlighted in bold).

| Variable | Effect | df | SS | | | *F*-statistic | *p*-value | Tukey |
| --- | --- | --- | --- | --- | --- | --- | --- | --- |
|  | |  | |  |  |  |  |  |
| *Acropora millepora* | |  | |  |  |  |  |  |
|  |  |  |  | | |  |  |  |
| 1^st^ Half | Model | 10, 35 | 0.1282 | | | 1.28 | 0.2955 |  |
|  | Error | 25 | 0.2512 | | |  |  |  |
|  | Temp | 1 | 0.0086 | | | 0.86 | 0.3625 |  |
|  | *p*CO_2_ | 2 | 0.0047 | | | 0.23 | 0.7938 |  |
|  | Colony | 5 | 0.0752 | | | 1.50 | 0.2265 |  |
|  | Temp x *p*CO_2_ | 2 | 0.0397 | | | 1.98 | 0.1597 |  |
|  |  |  |  | | |  |  |  |
| 2^nd^ Half | Model | 10, 33 | 0.0187 | | | 4.78 | **0.0009** |  |
|  | Error | 23 | 0.0090 | | |  |  |  |
|  | Temp | 1 | 0.0003 | | | 0.67 | 0.4203 |  |
|  | *p*CO_2_ | 2 | 0.0037 | | | 9.45 | **0.0010** | 382=60 > 607=741 |
|  | Colony | 5 | 0.0021 | | | 5.25 | **0.0023** | 3=2=4=5=1 > 2=4=5=1=6 |
|  | Temp x *p*CO_2_ | 2 | 0.0003 | | | 0.76 | 0.4769 |  |
|  | |  | |  |  |  |  |  |
| *Pocillopora damicornis* | |  | |  |  |  |  |  |
|  | |  | |  |  |  |  |  |
| 1^st^ Half | Model | 10, 35 | 73.7080 | | | 20.04 | **<0.0001** |  |
|  | Error | 25 | 9.1967 | | |  |  |  |
|  | Temp | 1 | 0.0687 | | | 0.19 | 0.6694 |  |
|  | *p*CO_2_ | 2 | 5.7149 | | | 7.77 | **0.0024** |  |
|  | Colony | 5 | 54.9847 | | | 29.89 | **<0.0001** | 4=6 > 6=5 > 2=3=1 |
|  | Temp x *p*CO_2_ | 2 | 12.9417 | | | 17.59 | **<0.0001** |  |
|  |  |  |  | | |  |  |  |
| 2^nd^ Half | Model | 10, 34 | 0.0684 | | | 3.82 | **0.0035** |  |
|  | Error | 24 | 0.0430 | | |  |  |  |
|  | Temp | 1 | 0.0073 | | | 4.06 | 0.0551 |  |
|  | *p*CO_2_ | 2 | 0.0106 | | | 2.96 | 0.0709 |  |
|  | Colony | 5 | 0.0388 | | | 4.33 | **0.0060** | 4=2=6=5 > 2=6=5=1=3 |
|  | Temp x *p*CO_2_ | 2 | 0.0106 | | | 2.95 | 0.0716 |  |
|  | |  | |  |  |  |  |  |
| *Montipora monasteriata* | |  | |  |  |  |  |  |
|  |  |  |  | | |  |  |  |
| 1^st^ Half | Model | 10, 35 | 0.2514 | | | 3.55 | **0.0049** |  |
|  | Error | 25 | 0.1768 | | |  |  |  |
|  | Temp | 1 | 0.0348 | | | 4.91 | **0.0360** | 29.0 > 26.5 |
|  | *p*CO_2_ | 2 | 0.0128 | | | 0.91 | 0.4166 |  |
|  | Colony | 5 | 0.1687 | | | 4.77 | **0.0034** | 6=4=2=1 > 4=2=1=5 > 2=1=5=3 |
|  | Temp x *p*CO_2_ | 2 | 0.0351 | | | 2.48 | 0.1042 |  |
|  |  |  |  | | |  |  |  |
| 2^nd^ Half | Model | 10, 35 | 0.5361 | | | 5.71 | **0.0002** |  |
|  | Error | 25 | 0.2348 | | |  |  |  |
|  | Temp | 1 | 0.0005 | | | 0.05 | 0.8208 |  |
|  | *p*CO_2_ | 2 | 0.0396 | | | 2.11 | 0.1427 |  |
|  | Colony | 5 | 0.4800 | | | 10.22 | **<0.0001** | 6=4 > 4=2 > 2=5=1=3 |
|  | Temp x *p*CO_2_ | 2 | 0.0160 | | | 0.85 | 0.4379 |  |
|  | |  | |  |  |  |  |  |
| *Turbinaria reniformis* | |  | |  |  |  |  |  |
|  | |  | |  |  |  |  |  |
| 1^st^ Half | Model | 10, 35 | 0.1838 | | | 4.51 | **0.0011** |  |
|  | Error | 25 | 0.1020 | | |  |  |  |
|  | Temp | 1 | 0.0024 | | | 0.59 | 0.4492 |  |
|  | *p*CO_2_ | 2 | 0.0086 | | | 1.06 | 0.3618 |  |
|  | Colony | 5 | 0.1619 | | | 7.94 | **0.0001** | 5=3=4=1=2 > 6 |
|  | Temp x *p*CO_2_ | 2 | 0.0109 | | | 1.34 | 0.2812 |  |
|  |  |  |  | | |  |  |  |
| 2^nd^ Half | Model | 10, 34 | 0.1828 | | | 2.89 | **0.0160** |  |
|  | Error | 24 | 0.1516 | | |  |  |  |
|  | Temp | 1 | 0.0125 | | | 1.97 | 0.1729 |  |
|  | *p*CO_2_ | 2 | 0.0339 | | | 2.68 | 0.0887 |  |
|  | Colony | 5 | 0.1308 | | | 4.14 | **0.0075** | 5=3=2=1=4 > 2=1=4=6 |
|  | Temp x *p*CO_2_ | 2 | 0.0044 | | | 0.35 | 0.7094 |  |

df = degrees of freedom, SS = sum of squares of the effects
