# Supplementary material for: Coral Energy Reserves and Calcification in a High-CO2 World at Two Temperatures
Source: PLoS One. 2013 Oct 11;8(10):e75049. doi: 10.1371/journal.pone.0075049 (PMC3795744; doi:10.1371/journal.pone.0075049)
Supplement: Table S3 — Results of 16 two-way ANOVAs for average soluble lipid, animal soluble protein, animal soluble carbohydrate concentrations, and tissue biomass. (DOCX) [file pone.0075049.s003.docx]

**Coral energy reserves and calcification in a high-CO_2_ world at two temperatures**

Verena Schoepf^1^, Andréa G. Grottoli^1^, Mark E. Warner^2^, Wei-Jun Cai^3,*^, Todd F. Melman^4^, Kenneth D. Hoadley^2^, D. Tye Pettay^2^, Xinping Hu^3,†^, Qian Li^3,‡^, Hui Xu^3,∆^, Yongchen Wang^3^, Yohei Matsui^1^, Justin H. Baumann^1^

**Author affiliations**

1. School of Earth Sciences, The Ohio State University, Columbus, OH, United States
2. School of Marine Science and Policy, University of Delaware, Lewes, DE, United States
3. Department of Marine Sciences, University of Georgia, Athens, GA, United States
4. Reef Systems Coral Farm, New Albany, OH, United States

* Present address: School of Marine Science and Policy, University of Delaware, Newark, DE, United States

† Present address: Department of Physical and Environmental Sciences, Texas A&M University, Corpus Christi, TX, United States

‡ Present address: State Key Laboratory of Marine Environmental Science, Xiamen University, Xiamen, China

∆ Present address: Department of Ocean Science and Engineering, Zhejiang University, Hangzhou, China

**Corresponding author**

Verena Schoepf

E-mail: schoepf.4@osu.edu

**Supplemental Table S3.** ***Results of 16 two-way ANOVAs for average soluble lipid, animal soluble protein, animal soluble carbohydrate concentrations, and tissue biomass.*** Four species (*Acropora millepora, Pocillopora damicornis, Montipora monasteriata, Turbinaria reniformis*) were compared at three *p*CO_2_ concentrations (382, 607, 741 μatm) and two temperature levels (26.5, 29.0°C) with colony as a random factor. Post hoc Tukey tests were used when main effects were significant. Effects were considered significant when *p*≤0.05 (highlighted in bold).

| Variable | Effect | | | | df | | SS | | | *F*-  statistic | *p*-value | | Tukey | |
| --- | --- | --- | --- | --- | --- | --- | --- | --- | --- | --- | --- | --- | --- | --- |
| *Acropora millepora* | |  | | |  | |  | | |  |  | |  | |
| Lipid | Model | | | | 10, 35 | | 0.0229 | | | 3.54 | **0.0050** | |  | |
|  | Error | | | | 25 | | 0.0162 | | |  |  | |  | |
|  | Temp | | | | 1 | | 0.0027 | | | 4.13 | 0.0529 | |  | |
|  | *p*CO_2_ | | | | 2 | | 0.0068 | | | 5.28 | **0.0123** | | 607=741 > 741=382 | |
|  | Colony | | | | 5 | | 0.0120 | | | 3.69 | **0.0122** | | 6=2=5=4=3 > 2=5=4=3=1 | |
|  | Temp x *p*CO_2_ | | | | 2 | | 0.0015 | | | 1.12 | 0.3423 | |  | |
|  |  | | | |  | |  | | |  |  | |  | |
| Protein | Model | | | | 10, 35 | | 0.1065 | | | 3.07 | **0.0112** | |  | |
|  | Error | | | | 25 | | 0.0869 | | |  |  | |  | |
|  | Temp | | | | 1 | | 0.0000 | | | 0.00 | 0.9471 | |  | |
|  | *p*CO_2_ | | | | 2 | | 0.0499 | | | 7.18 | **0.0034** | |  | |
|  | Colony | | | | 5 | | 0.0110 | | | 0.63 | 0.6752 | |  | |
|  | Temp x *p*CO_2_ | | | | 2 | | 0.0456 | | | 6.56 | **0.0051** | |  | |
|  |  | | | |  | |  | | |  |  | |  | |
| Carbohydrate | Model | | | | 10, 34 | | 0.0001 | | | 4.78 | **0.0008** | |  | |
|  | Error | | | | 24 | | 0.0000 | | |  |  | |  | |
|  | Temp | | | | 1 | | 0.0000 | | | 13.49 | **0.0012** | | 26.5 > 29.0 | |
|  | *p*CO_2_ | | | | 2 | | 0.0000 | | | 9.76 | **0.0008** | | 382=741 > 607 | |
|  | Colony | | | | 5 | | 0.0000 | | | 3.15 | **0.0251** | | 4=2=1=5=6 > 1=5=6=3 | |
|  | Temp x *p*CO_2_ | | | | 2 | | 0.0000 | | | 0.00 | 0.9991 | |  | |
|  |  | | | |  | |  | | |  |  | |  | |
| Tissue Biomass | Model | | | | 10, 35 | | 9.0170 | | | 0.98 | 0.4864 | |  | |
|  | Error | | | | 25 | | 48.6317 | | |  |  | |  | |
|  | Temp | | | | 1 | | 0.0319 | | | 0.02 | 0.8991 | |  | |
|  | *p*CO_2_ | | | | 2 | | 11.4659 | | | 2.95 | 0.0709 | |  | |
|  | Colony | | | | 5 | | 3.9026 | | | 0.40 | 0.8433 | |  | |
|  | Temp x *p*CO_2_ | | | | 2 | | 3.6165 | | | 0.93 | 0.4079 | |  | |
| *Pocillopora damicornis* | | |  |  | | |  | | |  |  | |  | |
| Lipid | Model | | | | 10, 35 | | 0.0591 | | | 4.21 | **0.0017** | |  | |
|  | Error | | | | 25 | | 0.0351 | | |  |  | |  | |
|  | Temp | | | | 1 | | 0.0006 | | | 0.41 | 0.5261 | |  | |
|  | *p*CO_2_ | | | | 2 | | 0.0146 | | | 5.22 | **0.0128** | | 607=741 > 741=382 | |
|  | Colony | | | | 5 | | 0.0411 | | | 5.86 | **0.0010** | | 1=2=3=4=5 > 3=4=5=6 | |
|  | Temp x *p*CO_2_ | | | | 2 | | 0.0028 | | | 0.98 | 0.3887 | |  | |
|  |  | | | |  | |  | | |  |  | |  | |
| Protein | Model | | | | 10, 35 | | 0.0101 | | | 0.43 | 0.9203 | |  | |
|  | Error | | | | 25 | | 0.0595 | | |  |  | |  | |
|  | Temp | | | | 1 | | 0.0009 | | | 0.37 | 0.5469 | |  | |
|  | *p*CO_2_ | | | | 2 | | 0.0030 | | | 0.63 | 0.5415 | |  | |
|  | Colony | | | | 5 | | 0.0050 | | | 0.42 | 0.8331 | |  | |
|  | Temp x *p*CO_2_ | | | | 2 | | 0.0013 | | | 0.27 | 0.7633 | |  | |
|  |  | | | |  | |  | | |  |  | |  | |
| Carbohydrate | Model | | | | 10, 35 | | 0.0000 | | | 0.49 | 0.8811 | |  | |
|  | Error | | | | 25 | | 0.0000 | | |  |  | |  | |
|  | Temp | | | | 1 | | 0.0000 | | | 0.00 | 0.9672 | |  | |
|  | *p*CO_2_ | | | | 2 | | 0.0000 | | | 0.75 | 0.4824 | |  | |
|  | Colony | | | | 5 | | 0.0000 | | | 0.63 | 0.6817 | |  | |
|  | Temp x *p*CO_2_ | | | | 2 | | 0.0000 | | | 0.13 | 0.8780 | |  | |
|  |  | | | |  | |  | | |  |  | |  | |
| Tissue Biomass | Model | | | | 10, 35 | | 0.0487 | | | 1.11 | 0.3959 | |  | |
|  | Error | | | | 25 | | 0.1102 | | |  |  | |  | |
|  | Temp | | | | 1 | | 0.0044 | | | 1.00 | 0.3257 | |  | |
|  | *p*CO_2_ | | | | 2 | | 0.0081 | | | 0.92 | 0.4101 | |  | |
|  | Colony | | | | 5 | | 0.0281 | | | 1.27 | 0.3066 | |  | |
|  | Temp x *p*CO_2_ | | | | 2 | | 0.0081 | | | 0.92 | 0.4124 | |  | |
| *Montipora monasteriata* | | |  |  | | |  | | |  |  | |  | |
| Lipid | Model | | | | 10, 35 | | 0.0031 | | | 0.89 | 0.5544 | |  | |
|  | Error | | | | 25 | | 0.0087 | | |  |  | |  | |
|  | Temp | | | | 1 | | 0.0003 | | | 0.79 | 0.3816 | |  | |
|  | *p*CO_2_ | | | | 2 | | 0.0011 | | | 1.57 | 0.2280 | |  | |
|  | Colony | | | | 5 | | 0.0017 | | | 0.96 | 0.4582 | |  | |
|  | Temp x *p*CO_2_ | | | | 2 | | 0.0001 | | | 0.08 | 0.9254 | |  | |
|  |  | | | |  | |  | | |  |  | |  | |
|  |  | | | |  | |  | | |  |  | |  | |
|  |  | | | |  | |  | | |  |  | |  | |
|  |  | | | |  | |  | | |  |  | |  | |
|  |  | | | |  | |  | | |  |  | |  | |
| Protein | Model | | | | 10, 35 | | 0.2040 | | | 6.67 | **<0.0001** | |  | |
|  | Error | | | | 25 | | 0.0765 | | |  |  | |  | |
|  | Temp | | | | 1 | | 0.0135 | | | 4.42 | **0.0457** | |  | |
|  | *p*CO_2_ | | | | 2 | | 0.0070 | | | 1.15 | 0.3322 | |  | |
|  | Colony | | | | 5 | | 0.1047 | | | 6.85 | **0.0004** | | 6=4=3 > 4=3=2=1=5 | |
|  | Temp x *p*CO_2_ | | | | 2 | | 0.0787 | | | 12.86 | **0.0001** | |  | |
|  |  | | | |  | |  | | |  |  | |  | |
| Carbohydrate | Model | | | | 10, 35 | | 0.0001 | | | 2.45 | **0.0337** | |  | |
|  | Error | | | | 25 | | 0.0001 | | |  |  | |  | |
|  | Temp | | | | 1 | | 0.0000 | | | 3.25 | 0.0834 | |  | |
|  | *p*CO_2_ | | | | 2 | | 0.0000 | | | 0.49 | 0.6201 | |  | |
|  | Colony | | | | 5 | | 0.0001 | | | 2.86 | **0.0354** | | 6=4=5=3=2 > 5=3=2=1 | |
|  | Temp x *p*CO_2_ | | | | 2 | | 0.0000 | | | 2.98 | 0.0690 | |  | |
|  |  | | | |  | |  | | |  |  | |  | |
| Tissue Biomass | Model | | | | 10, 31 | | 48.0895 | | | 2.24 | 0.0572 | |  | |
|  | Error | | | | 21 | | 45.0394 | | |  |  | |  | |
|  | Temp | | | | 1 | | 0.1734 | | | 0.08 | 0.7789 | |  | |
|  | *p*CO_2_ | | | | 2 | | 9.9874 | | | 2.33 | 0.1221 | |  | |
|  | Colony | | | | 5 | | 37.6842 | | | 3.51 | **0.0183** | |  | |
|  | Temp x *p*CO_2_ | | | | 2 | | 8.5097 | | | 1.98 | 0.1625 | |  | |
| *Turbinaria reniformis* | | | | |  |  |  |  | | | |  |  |  |
| Lipid | Model | | | | 10, 35 | | 0.0034 | | 0.88 | | 0.5612 | |  | |
|  | Error | | | | 25 | | 0.0096 | |  | |  | |  | |
|  | Temp | | | | 1 | | 0.0003 | | 0.75 | | 0.3950 | |  | |
|  | *p*CO_2_ | | | | 2 | | 0.0004 | | 0.48 | | 0.6220 | |  | |
|  | Colony | | | | 5 | | 0.0026 | | 1.36 | | 0.2715 | |  | |
|  | Temp x *p*CO_2_ | | | | 2 | | 0.0001 | | 0.15 | | 0.8642 | |  | |
|  |  | | | |  | |  | |  | |  | |  | |
| Protein | Model | | | | 10, 34 | | 0.0520 | | 2.22 | | 0.0537 | |  | |
|  | Error | | | | 24 | | 0.0564 | |  | |  | |  | |
|  | Temp | | | | 1 | | 0.0036 | | 1.54 | | 0.2262 | |  | |
|  | *p*CO_2_ | | | | 2 | | 0.0167 | | 3.56 | | **0.0443** | |  | |
|  | Colony | | | | 5 | | 0.0190 | | 1.61 | | 0.1943 | |  | |
|  | Temp x *p*CO_2_ | | | | 2 | | 0.0100 | | 2.10 | | 0.1444 | |  | |
|  |  | | | |  | |  | |  | |  | |  | |
| Carbohydrate | Model | | | | 10, 35 | | 0.0001 | | 0.86 | | 0.5830 | |  | |
|  | Error | | | | 25 | | 0.0002 | |  | |  | |  | |
|  | Temp | | | | 1 | | 0.0000 | | 0.66 | | 0.4233 | |  | |
|  | *p*CO_2_ | | | | 2 | | 0.0000 | | 0.21 | | 0.8137 | |  | |
|  | Colony | | | | 5 | | 0.0000 | | 0.93 | | 0.4806 | |  | |
|  | Temp x *p*CO_2_ | | | | 2 | | 0.0000 | | 1.43 | | 0.2592 | |  | |
|  |  | | | |  | |  | |  | |  | |  | |
| Tissue Biomass | Model | | | | 10, 35 | | 101.41 | | 2.41 | | **0.0362** | |  | |
|  | Error | | | | 25 | | 105.14 | |  | |  | |  | |
|  | Temp | | | | 1 | | 1.0452 | | 0.25 | | 0.6225 | |  | |
|  | *p*CO_2_ | | | | 2 | | 4.7408 | | 0.56 | | 0.5762 | |  | |
|  | Colony | | | | 5 | | 72.994 | | 3.47 | | **0.0161** | | 3=4=2=1 > 4=2=1=5=6 | |
|  | Temp x *p*CO_2_ | | | | 2 | | 22.628 | | 2.69 | | 0.0875 | |  | |

df = degrees of freedom, SS = sum of squares of the effects
